# Emerging Insight Into the Role of Circadian Clock Gene BMAL1 in Cellular Senescence

**DOI:** 10.3389/fendo.2022.915139

**Published:** 2022-06-06

**Authors:** Wenqian Zhang, Yuan Xiong, Ranyang Tao, Adriana C. Panayi, Bobin Mi, Guohui Liu

**Affiliations:** ^1^ Department of Orthopaedics, Union Hospital, Tongji Medical College, Huazhong University of Science and Technology, Wuhan, China; ^2^ Hubei Province Key Laboratory of Oral and Maxillofacial Development and Regeneration, Wuhan, China; ^3^ Division of Plastic Surgery, Brigham and Women’s Hospital and Harvard Medical School, Boston, MA, United States

**Keywords:** BMAL1, aging, cellular senescence, oxidative stress, metabolism, genotoxic stress

## Abstract

Cell senescence is a crucial process in cell fate determination and is involved in an extensive array of aging-associated diseases. General perceptions and experimental evidence point out that the decline of physical function as well as aging-associated diseases are often initiated by cell senescence and organ ageing. Therefore, regulation of cell senescence process can be a promising way to handle aging-associated diseases such as osteoporosis. The circadian clock regulates a wide range of cellular and physiological activities, and many age-linked degenerative disorders are associated with the dysregulation of clock genes. BMAL1 is a core circadian transcription factor and governs downstream genes by binding to the E-box elements in their promoters. Compelling evidence has proposed the role of BMAL1 in cellular senescence and aging-associated diseases. In this review, we summarize the linkage between BMAL1 and factors of cell senescence including oxidative stress, metabolism, and the genotoxic stress response. Dysregulated and dampened BMAL1 may serve as a potential therapeutic target against aging- associated diseases.

## Introduction

Aging is a universal and intrinsic process with deleterious effects including gradual accumulation of physical and molecular dysfunction, tissue degradation and diminished organ function, which increases risk of developing age-related diseases and ultimately leads to death (1, 2). Cellular senescence, one of the hallmarks of aging, is a state of irreversible cell-cycle arrest in response to a variety of cellular stresses including genotoxic stress, and oxidative and telomere dysfunction (3, 4), and shows strong linkage to physiological processes such as embryogenesis, tissue repair, tumour suppression and organismal ageing (5). Cellular senescence can be divided into replicative senescence and stress induced premature senescence. Replicative senescence is a telomere-dependent senescence, while premature senescence results from all types of cellular stress except telomere shortening (6). Compelling evidence points towards an interplay between cellular senescence and pathological processes in many types of cells and tissues. Almost all multicellular organisms display features of aging, which lead to the generation of numerous chronic and age-related pathologies and progressive loss in tissue and organ function over time. Cellular senescence is related to a variety of aging-associated diseases, including nonalcoholic fatty liver disease, diabetes, pulmonary hypertension, osteoarthritis and tumorigenesis (7). Moreover, the observation that elimination of senescent cells is largely beneficial and has been considered as one of the main therapeutic strategies for aging-associated diseases (5). The elimination of naturally occurring senescent cells has been reported to ameliorate the age-related deterioration of several organs and tissues and to extend lifespan in mice (8). Hence, research on the mechanisms of aging and cellular senescence is significant in the face of a wide range of disease.

Circadian clock, established by cell-autonomous oscillators, generates 24-h rhythms in physiology and behavior in various organisms. Many cellular and physiological activities are under the control of the circadian rhythm and there is emerging evidence that the circadian clock is intimately intertwined with the aging process (9). Besides, some studies also have pointed out that the circadian clock system is disrupted with aging (10–12). Alterations of the circadian clock have been reported in senescent cells (13) and stress-induced premature senescent cells (14), which showed a longer period and delayed phase compared to proliferative cells.

Brain and muscle arnt-like protein 1 (BMAL1), the core circadian gene, whose expression decreases in the natural aging process, has been shown to significantly affect the senescence process and governs many aspects of age-related pathology (15, 16). The most compelling evidence has demonstrated that mice deficient in BMAL1 have a significantly reduced lifespan and display early aging phenotypes including sarcopenia, cataracts, low subcutaneous fat, and organ shrinkage among others (17). Furthermore, overexpression of AHA-1, a homolog of mammalian BMAL1 protein, extends the lifespan in C. elegans (18).

Therefore, in this review, we summarized recent studies focusing on the effect of BMAL1 in aging and cellular senescence, highlighting its mechanisms in oxidative stress, metabolism, and the genotoxic stress response.

## BMAL1 and Circadian Rhythm

### A Brief Introduction of BMAL1

BMAL1, also referred to as Arntl, Arnt3, and MOP3, is a transcription factor that has a basic-helix-loop-helix (bHLH)/Per-Arnt-Sim (PAS) domain (15, 19). Ikeda et al. (19) firstly isolated and identified BMAL1 from the human brain cDNA library. According to their work, BMAL1 was originally characterized by its enrichment in the brain and the skeletal muscle, implying the significant role in these tissues (19).

Further research has identified the function of BMAL1 in the circadian rhythm and other complex physiologic properties. BMAL1 forms heterodimers with another bHLH/PAS protein, circadian locomotor output cycles kaput (CLOCK), and the complex drives transcription from E-box elements and regulates the circadian rhythm of a spectrum of gene expressions (20). Gene Ontology analysis of genes commonly occupied by BMAL1 shows enrichment for circadian circuits, as well as carbohydrate metabolism, lipid metabolism, and amino acid metabolism (21). Global BMAL1^−/−^mice display several severe phenotypic changes in the rhythmicity of behavior, metabolic activity and life span (17, 22).

### Role of BMAL1 in the Circadian Rhythm

The regulation effects in the circadian rhythm, the main function of BMAL1, links a wide range of cellular and physiological activities and is under extensive investigation.

**Figure 1:**
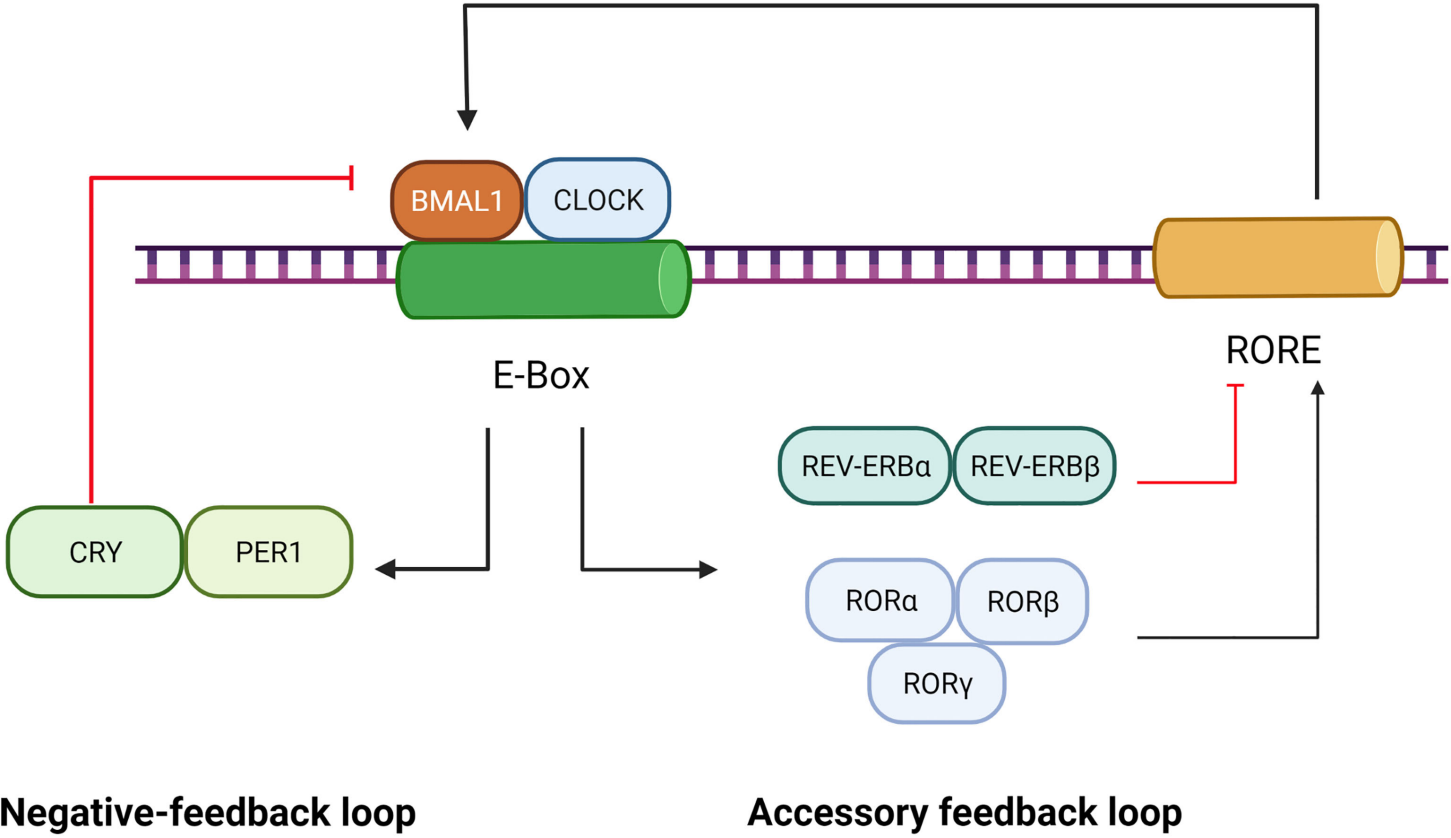
Role of BMAL1 in the circadian rhythm. The circadian rhythm involves a negative-feedback loop and an accessory feedback loop. In the negative-feedback loop, the CLOCK and BMAL1 form a heterodimer that activates the PER and CRY genes by directly interacting with the E-Box element. Subsequently, accumulation of PER/CRY protein complexes inhibits the expression of BMAL1/CLOCK. In the accessory feedback loop, the transcription of BMAL1/CLOCK is controlled by positive regulators (RORα, RORβ and RORγ) and negative regulators (REV-ERBα and REV-ERBβ). ClOCK and BMAl1, in turn, regulate the expression of RORs and REV-ERBs.

Circadian rhythms refer to the variations in physiology and behavior that peak and trough within the 24-hour timescale, and resides in the suprachiasmatic nucleus (SCN) of the hypothalamus (23). Circadian rhythms are driven by cell-autonomous oscillating circadian molecular clocks built upon molecular feedback loops (24) including the transcriptional negative-feedback loop and accessory feedback loop, which are summarized in **Figure 1**.

The transcriptional negative-feedback loop consists of a set of clock proteins that include core clock transcription factors BMAL1, CLOCK, transactivating promoter E-box elements of Period (Per1, Per2, and Per3), and cryptochrome (Cry1 and Cry2). The CLOCK and BMAL1 form heterodimers to activate the PER and CRY genes as well as a spectrum of clock-controlled genes (CCGs) by directly interacting with E-Box elements (nCACGTGn) present in gene promoters (25). Subsequently, accumulation of PER/CRY protein complexes inhibits the expression of BMAL1/CLOCK, resulting in rhythmic repression of their own transcription and of other CCGs (26).

The accessory feedback loop involves REV-ERBα/β (27) and RORα/β/γ (28). The nuclear receptor retinoid-related orphan receptors(RORs), such as RORα, was identified as an activator of Rev response element (RRE) box-containing clock genes such as BMAL1 (26). Reverse orientation c-erbA gene α(REV-ERBα), a widely expressed member of the orphan nuclear receptor family of proteins, is a strong repressor of BMAL1 transcription and a moderate repressor of CLOCK transcription, which couples antiphasic transcription cycles of negative and positive limb members with its paralog REV-ERBβ (29). In mammals, the accessory feedback loop controls the transcriptional regulation of CLOCK and BMAL1 (30), involving both positive regulators (RORα, RORβ and RORγ) and negative ones (REV-ERBα and REV-ERBβ). ClOCK and BMAl1, in turn, regulate the expression of RORs and REV-ERBs (29, 31). Given that REV-ERBα, REV-ERBβ, ROR-α, ROR-β and ROR-γ bind to a common response element—the ROR response element,—their intrinsic repressive and inductive activities, are believed to establish the rhythmic expression of target genes such as BMAL1 (28).

As a core clock transcription factor, BMAL1 plays a critical role in governing the molecular clock, which has already become the key object of study for circadian rhythm and functional research. BMAL1 is the core orchestrator of the molecular clock, and the only single clock gene deletion that results in complete ablation of all rhythms (25). BMAL1 also mediates the nuclear translocation (32) and site-specific phosphorylation/degradation of CLOCK (33), both of which are associated with the activation of responsive circadian promoters.

Numerous studies involving global temporal gene-expression profiling revealed that about 10% of the mammalian transcriptome shows daily rhythm in steady-state mRNA levels including many key regulators of cell cycle, DNA repair, genotoxic stress response, and metabolism (34), which suggests the significant role of the circadian rhythm. Biological clocks regulate a wide range of cellular and physiological activities, and many age-associated disorders are related to the dysregulation of the circadian rhythm (35). As the key factor of the circadian rhythm, the importance of BMAL1 in cellular senescence and aging-associated diseases needs to be investigated.

Although ample evidence clearly demonstrated the role of the circadian clock in cellular senescence and aging- associated diseases, the precise molecular mechanisms of BMAL1 action in aging are a subject for further studies. Previous studies have identified that circadian proteins could be responsible for aging through the regulation of metabolism, genotoxic stress response and reactive oxygen species (ROS) homeostasis (12, 36).

## Role of BMAL1 in Oxidative Stress

Oxidative stress refers to an imbalance between oxidants and antioxidants in favor of oxidants, which leads to a disruption of redox signaling and molecular damage (37). Oxidative stress is a cell damage-related process that causes damage to proteins, lipids, and DNA in the cells, leading to cell death and subsequent tissue dysfunction. Reactive oxygen species (ROS), a major product of oxidative stress, induce cell senescence involving disruption of proteostasis, alteration of genomic stability, the response to DNA damage, epigenetic regulation, and activation of the tumor suppression pathway (38).

A major characteristic of aging is an increase in oxidative stress, which is considered one of the molecular mechanisms underlying the occurrence and development of a variety of age-related pathologies. In aging, oxidant production from several sources is increased, whereas antioxidant enzymes, the primary line of defense, are decreased. Indeed, oxidative stress has been implicated in various age-related pathologies including cancers (39), neurodegenerative diseases (40), diabetes (41), and cardiovascular conditions (42).

Many factors are involved in the occurrence and regulation of oxidative stress. Strong evidence supports that mitochondrial dysfunction leads to oxidative stress, which is involved in age-related deteriorations in part through oxidative damage (43). Furthermore, oxidative stress is under the regulation of certain signaling pathways. The Nrf2 pathway and the NFκB pathway are considered master switch systems of oxidative stress in cells of higher organisms (37). Numerous studies have linked oxidative stress and aging with the disruptions of the aforementioned pathways (44, 45).

Compelling evidence points toward an interplay between the circadian clock and the cellular redox status (46). Circadian clocks maintain ROS at physiological levels and protect organisms from oxidative stress (47).

The function of BMAL1 is not only limited to core clock gene regulation and oscillation, but also involved in maintaining redox homeostasis and promoting cell survival against oxidative stress. Studies have pointed out that the cellular abundance of BMAL1 is a prerequisite for promoting antioxidant defense to protect cells against aging or oxidative stress (35). Global deletion of BMAL1 produces an advanced aging phenotype by increasing oxidative stress (48). In addition, there also exists evidence that BMAL1 regulates ROS in multiple independent tissue types. Deletion of BMAL1 in the brain contributes to oxidative stress-induced neurodegeneration and astrogliosis (9). Deletion of BMAL1 in the pancreas contributes to a diabetic phenotype due to oxidative stress-induced β-cell failure (49). However, the accelerated aging phenotype, produced in BMAL1 knockouts, can be rescued by using glutathione precursor NAC (50). Taken together, this evidence highlights the significance of oxidative stress in BMAL1-deficient-related aging phenotype.

Given the aforementioned mechanisms of oxidative stress, this review will summarize the role of BMAL1 in oxidative stress concerning the Nrf2 pathway, the NFκB pathway and mitochondrial dysfunction.

### Deficiency of BMAL1 Leads to Oxidative Stress Through the Nrf2 Pathway

The nuclear factor erythroid 2-related factor (Nrf2) is part of one of the most important pathways for intracellular endogenous antioxidant stress. Nrf2 is a basic leucine zipper (bZIP) transcription factor, regulating the expression of antioxidant proteins and protecting cells against oxidative damage (51). Nrf2 is normally inactive in the cytoplasm and binds to Kelch-like ECH-associated protein 1 (Keap1), which sequesters Nrf2, facilitating its degradation. On activation by oxidative stress, Nrf2 dissociates from Keap1 and translocates into the nucleus through the oxidation or covalent modification of Keap1 cysteine residues (52). In the nucleus, Nrf2 binds to the antioxidant response elements (AREs) and promotes the expression of antioxidant genes, such as Hmox1, Gsr, and Nqo1 (51). A multitude of publications has shown that Nrf2 is an essential regulator of longevity. It has been reported that activation of Nrf2 prolongs the life span of mice and Caenorhabditis elegans (53, 54).

One study has pointed out that Nrf2 creates direct and reciprocal coupling between the redox state and the circadian mechanism. Previous studies have identified Nrf2 as one of the clock-controlled targets in pancreatic β cells (49) and mouse lungs (55), where mRNA levels of Nrf2, as well as several of its target genes, have been reported to show diurnal variation.

Nrf2 and BMAL1 comprise an interlocking loop that integrates cellular redox signals into circadian timekeeping. On the one hand, BMAL1 participates in the regulation of the Nrf2 pathway. BMAL1, together with CLOCK construct a complex, exerting temporal control of the E-box element in the Nrf2 gene promoter and positively regulating Nrf2 transcription. Nrf2 protein accumulates in a circadian manner and drives oscillations of ARE-regulated antioxidant genes. Mutation of the E-box sequence in the Nrf2 promoter completely abolishes its induction by CLOCK/BMAL1 complexes (55). On the other hand, Nrf2 represses CLOCK/BMAL1-mediated transcription indirectly through the regulation of Cry2 expression. Nrf2 specifically attaches to part of the gene of the clock protein CRY2. As levels of CRY rise, they repress CLOCK/BMAL1-regulated E-box transcription and switch off their production (56). Thus, these molecules rise and fall throughout the day to drive circadian rhythms just as a pendulum swings back and forth to keep a clock ticking.

**Figure 2:**
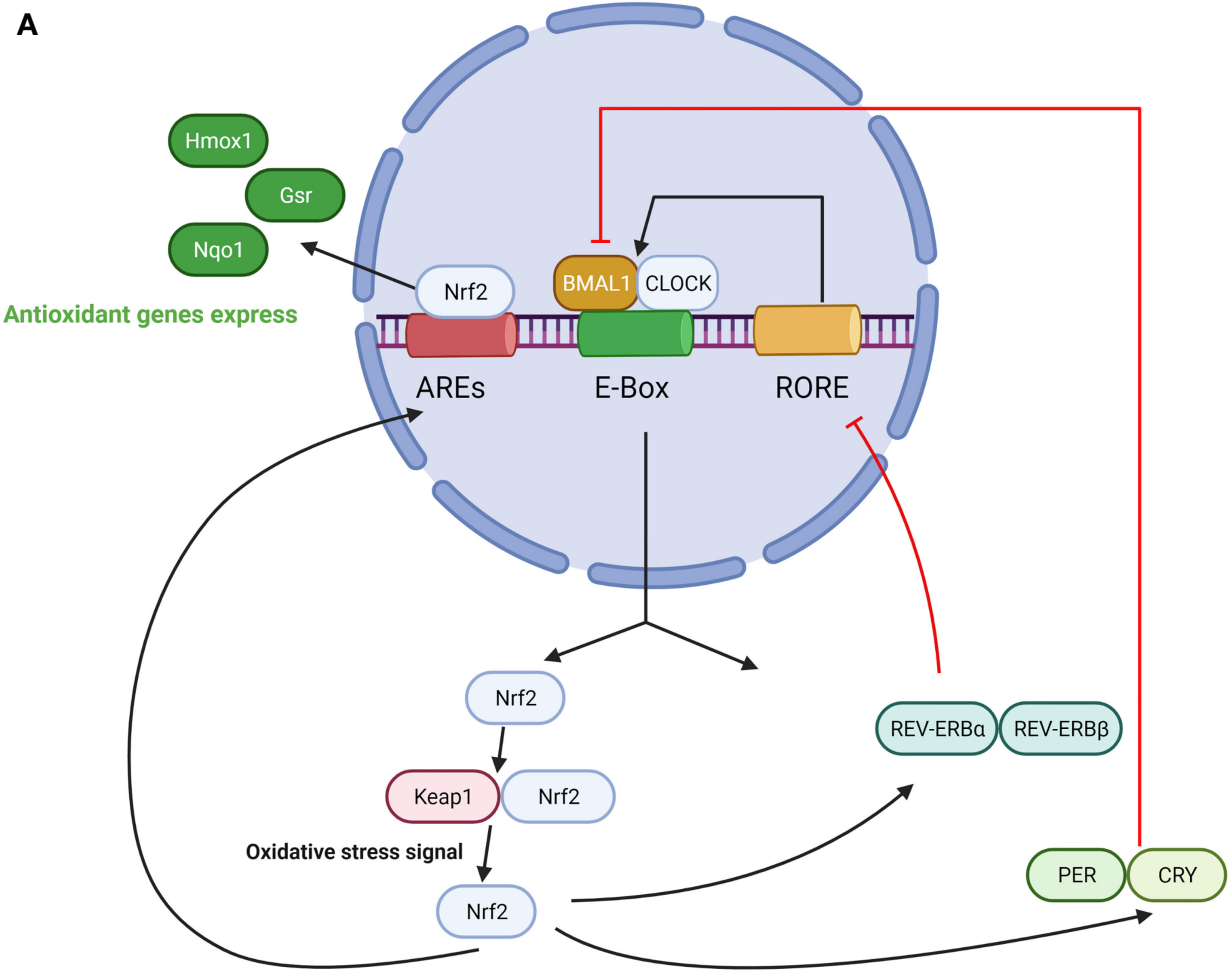
**(A)** The role of BMAL1 in the Nrf2 pathway. CLOCK/BMAL1 complexes exert temporal control on the E-box element in the Nrf2 gene promoter and positively regulate Nrf2 transcription. Nrf2 protein accumulates in a circadian manner and drives oscillations of ARE-regulated antioxidant genes. On the other hand, Nrf2 regulates the expression of PER, CRY, REV-ERBα/β, which represses CLOCK/BMAL1-regulated E-box transcription and switches off their production. **(B)** The role of BMAL1 in the NFκB pathway. On one hand, NFκB activation is under the regulation of a loop represented by the clock genes BMAL1/CLOCK, and their transcriptional positive and negative regulators REV-ERBα and RORα. Activation of this pathway leads to enhanced NAD+ levels and SIRT1 deacetylase activity, which in turn inactivates NFκB and reduces the binding ability to DNA. CLOCK upregulates NFκB–mediated transcription by increasing p65 phosphorylation and acetylation in the absence of BMAL1, and BMAL1 downregulates the CLOCK-dependent modulation of NFκB activation. On the other hand, NFκB antagonizes transcription of the CLOCK/BMAL1 target genes by directly binding to the promoters of the core clock repressors PER and CRY, which leads to highly specific transcriptional repression of CLOCK/BMAL1.

Overall, the biological clock protein BMAL1 is a significant component of the regulation of the Nrf2-mediated antioxidant protective response in maintaining ROS homeostasis within the microenvironment for cellular protection and health. The role of BMAL1 in the Nrf2 pathway is summarized in **Figure 2**.

### Deficiency of BMAL1 Leads to Oxidative Stress *via* the NFκB Pathway

The nuclear factor kappa B (NFκB) is one of the principal transcriptional regulators, as well as a cytosolic sensor of multiple dangerous signals including oxidative stress, a process which activates and promotes its nuclear translocation and DNA binding (57). In mammals, the major cellular form of NFκB is a heterodimer consisting of the DNA binding subunit p50 and the transactivator p65. Normally, NFκB is bound to its inhibitor IκB, which retains NFκB in the cytoplasm. Upon stimulation by various NFκB activating signals, ligand recognition results in the recruitment of various adapter proteins, triggering the activation of the IκB kinase (IKK) complex followed by IκBα phosphorylation and subsequent degradation. IκB undergoes phosphorylation and degradation and frees NFκB, which is then translocated into the nucleus to bind to its consensus sites in promoters of specific genes, activating their expression (58).

NFκB characterizes the aging process; mounting evidence supports that NFκB content and its DNA binding increase with age (59, 60). Furthermore, NFκB signaling has been linked to cellular senescence. NFκB signaling is implicated in indoxyl sulfate‐induced cellular senescence *via* the ROS‐NFκB‐p53 pathway (61), further supporting a connection between the two conditions.

BMAL1 deficiency results in a misbalance in ROS generation/neutralization not only due to the dysregulation of downstream transcriptional anti- and pro-oxidant CLOCK/BMAL1 targets, but also through an NFκB–dependent mechanism (62). Accumulating evidence shows the presence of bidirectional links between BMAL1 and NFκB.

On the one hand, BMAL1 represses the activation of NFκB. NFκB activation is under the regulation of a loop represented by the clock genes BMAL1/CLOCK, and their transcriptional positive and negative regulators REV-ERBα and RORα (63, 64). Activation of this pathway leads to increased NAD^+^ levels and silent information regulator type 1 (SIRT1) deacetylase activity, which in turn inactivates NFκB and reduces its binding ability to DNA (65). In the aging process, CLOCK and BMAL1 gene expression reduce, which causes SIRT1/NAD^+^ dissociation and impedes SIRT1-dependent p65 subunit inhibition by deacetylation, resulting in enhanced NFκB transcriptional activity (66). The consequent enhanced NFκB binding to DNA promotes the expression of multiple proinflammatory and prooxidant molecules such as iNOS and TNFα, and pro-IL-1β, and is followed by subsequent mitochondrial impairment and oxidative stress, which are involved in the pathogenesis of most age-associated diseases (67). Another study elucidated further details about the indirect mechanism of BMAL1 in the regulation of NFκB through CLOCK. CLOCK upregulates NFκB–mediated transcription by increasing p65 phosphorylation and acetylation in the absence of BMAL1, whereas BMAL1 is crucial for reducing the scale of the inflammatory response and oxidative stress through downregulation of CLOCK-dependent modulation of NFκB activation (68).

On the other hand, NFκB also participates in the regulation of circadian proteins and circadian function. NFκB pathway activation results in suppression of BMAL1 expression and function (69). One study has reported that NFκB antagonizes transcription of CLOCK/BMAL1 target genes by directly binding to the promoters of the core clock repressors PER and CRY, which leads to a highly specific transcriptional repression signature at core clock genes (70). Another study reported that the RelB subunit of NFκB can act as a repressor of circadian transcription. In the presence of CLOCK, RelB physically interacts with BMAL1 to repress circadian gene expression at the promoter of the clock-controlled gene Dbp (71).

Collectively, these findings demonstrate bidirectional links between BMAL1 and NFκB. BMAL1 participates in the regulation of NFκB–dependent ROS generation/neutralization in the aging process. NFκB represses BMAL1 function. Given that NFκB activation may contribute to metabolic disease and aging in part through dysregulation of BMAL1 circadian systems, further study can take NFκB into consideration as a plausible target for therapeutic repression in anti-aging. The role of BMAL1 in the NFκB pathway is illustrated in **Figure 2**.

### Deficiency of BMAL1 Leads to Oxidative Stress Through Mitochondrial Dysfunction

Mitochondria are essential organelles involved in nutrient metabolism and energy homeostasis. In mitochondria, oxidation of glucose and fatty acids releases NADH and FADH2 that carry high-energy electrons to generate ATP through the electron transport chain (ETC) coupled to the oxidative phosphorylation (OXPHOS) of ADP. In the process of mitochondrial oxidative metabolism, an unavoidable side-product is ROS that results in detrimental protein and DNA modifications and cellular senescence as mentioned above.

In senescent cells, increased oxidative stress has been associated with the accumulation of dysfunctional mitochondria. Furthermore, senescent cells are characterized by changes in mitochondrial mass, membrane potential and mitochondrial morphology (72). Mitochondria are widely considered to play a significant role in the aging process, as they are major intracellular sources of ROS as well as the primary targets of ROS oxyradical attack (73). Accumulation of ROS results in impairment of mitochondrial function, which in turn contributes to a vicious cycle with a rise in mitochondrial ROS production and triggers subsequent serious cellular senescence and cellular death (74).

A rapidly growing body of research suggests a cross-talk between BMAL1 and mitochondrial function, including mitochondrial dynamics and mitochondrial respiration. Mitochondrial dynamics and oxidative metabolism are expressed in a daily BMAL1- dependent manner (75). *In vivo* studies have revealed that BMAL1 mutation disrupts mitochondrial dynamics and oxidative metabolism, ultimately resulting in metabolic dysregulation and oxidative damage (18).

BMAL1 is critical for the maintenance of normal mitochondrial structure and mitochondrial dynamics. Mitochondrial fission, fusion, and mitophagy, collectively referred to as mitochondrial dynamics, are the processes through which mitochondria achieve morphological and functional adaptations to accommodate different metabolic states and energy demands. Mitochondrial dynamics have drawn much attention for clear roles in mitochondrial quality control and in the development of aging-related diseases (76).

Mitochondrial dynamics are transcriptional targets of the circadian regulator BMAL1 and exhibit a metabolic rhythm in sync with diurnal bioenergetic demands (18).

There is emerging evidence that BMAL1 loss-of-function causes mitochondrial structure and mitochondrial dynamics incapable of adapting to different nutrient conditions, accompanied by diminished respiration and elevated oxidative stress. An abnormal mitochondrial structure has been observed in BMAL1-deficient cells. Ultrastructural changes including swollen mitochondria with cristae damage and irregular shape have been reported in BMAL1-deficient hepatocytes (18), BMAL1 knockdown β-cell (77) and cardiomyocytes (78). Mitochondrial morphology impairment, ATP synthesis reduction, as well as oxidative stress product accumulation can further result in cell damage and apoptosis (79).

Disruption of mitochondrial dynamics proteins in BMAL1-deficient cells also highlights the importance of BMAL1 in mitochondrial stability. BMAL1 deficiency results in reduced fusion gene (Mfn1, Mfn2) expression and increased fission/mitophagy protein (Fis1) expression in BMAL1 knockdown β-cells (77). However, research has shown reduced levels of fission/mitophagy proteins (Fis1, Pink1, and Drp1/phospho-Drp1 s616) as well as an increase in Mfn1 in BMAL1- knockout hepatocytes (18). Indeed, fusion proteins, such as Mfn1 and Mfn2, are required for normal mitochondrial metabolism and mitochondrial remodeling (80). Lacking Mfn1 or Mfn2 causes mitochondrial fragmentation as well as deficiency in mitochondrial structure and mitochondrial fusion (81). Fission/mitophagy proteins, such as Fis1, have been reported to mediate mitochondrial quality control (82) and transmit an apoptosis signal from the mitochondria to the endoplasmic reticulum (83). Although regulation may differ for each organ, these studies provide evidence that BMAL1 participates in the manipulation of mitochondrial dynamic genes and disruption of this process induces oxidative damage and metabolic dysregulation.

In addition to mitochondrial dynamics, there also exists evidence that points toward a link between BMAL1 and mitochondrial oxidative metabolism. Deletion of BMAL1 leads to upregulation of uncoupling proteins (Ucp2) with uncoupling of mitochondrial oxidative phosphorylation (OXPHOS), causing a decreased mitochondrial membrane potential (MMP) with reduced ROS clearance and antioxidant capacity (84).

Collectively, aforementioned studies reveal that synchronization of mitochondrial biogenesis and dynamics with nutrient status by BMAL1 is crucial to sustain energy homeostasis and health span. Deficiency of BMAL1 impairs normal mitochondrial structure, mitochondrial dynamics and mitochondrial metabolism, which account for subsequent oxidative stress as well as cellular senescence and death.

### Deficiency of BMAL1 Leads to Oxidative Stress Through NAD+ Regulation

NAD+ plays crucial role in intracellular redox reactions and works as a cofactor for some important enzymes, such as the DNA repair poly enzyme poly ADP-ribose polymerase (PARP) and the deacetylase Sirtuin 1 (SIRT1). Overexpression of nicotinamide phosphoribosyl transferase (NAMPT), the rate-limiting enzyme of the NAD+ salvage pathway, showed a protective effect against stress-induced premature senescence (85), demonstrating that a decline in NAD+ level can induce cellular senescence as well as organismal aging. The amount of NAD+ is altered with the aging process and shows great linkage to circadian clock, which is attributed to posttranslational modifications of acetylation and poly-ADP-ribosylation status of circadian clock proteins and decreases with aging (86). And one recent study has pointed that NAD+ levels can be a potential mechanism that links the circadian clock with aging (12).

On the one hand, the NAD^+^-driven pathway regulates circadian transcription patterns. Reduction in NAMPT-mediated NAD^+^ biosynthesis can lead to a more robust oscillation of clock target gene expression through releasing CLOCK : BMAL1 heterodimer from SIRT1-mediated suppression (87). NAD^+^ promotes BMAL1 transcription by SIRT1 through the deacetylation of PGC1α (88). Besides, another study has pointed that low NAD^+^ condition increased REB-ERBα/β and promoted PER2 to be retained in the cytoplasm through the NAD^+^/SIRT1 axis, which in turn repress the expression of BMAL1 gene (86).

On the other hand, BMAL1 regulates NAD^+^ levels. One study has observed that mice deficient in BMAL1 exhibited a significant reduction in NAD^+^ levels in live (87). BMAL1:CLOCK heterodimers bind to E-box elements of NAMPT to drive expression (89), thus participating in the relulation of NAD^+^.

NAD^+^ modulates the BAML1 and other circadian clock molecules, while NAMPT is under the control of the BMAL1. These facts demonstrate the bidirectional interactions between BMAL1 and NAD^+^, which is involved in oxidative stress and aging process.

## Role of BMAL1 in the Regulation of Nutrition Metabolism

The role of metabolic regulation in aging has been the focus of extensive research. There is emerging evidence that links longevity with metabolic parameters from different organs (90). For example, in glucose metabolism, the process of glycation has been considered to cause macromolecular damage and biochemical changes that occur in aging and age-related disorders, supported by the fact that several age-related diseases show symptoms manifested by hyperglycemia (91). In fatty acid metabolism, studies have revealed a positive correlation between longevity and sphingomyelin levels as well as a negative correlation between longevity and the levels of triacylglycerols containing polyunsaturated fatty acid (PUFA) side chains and by-products of inflammatory processes (92). In amino acid metabolism, longevity also negatively correlates with the hepatic concentrations of tryptophan degradation products and with the hepatic levels of enzymatic cofactors involved in amino acid metabolism (93). Nutrient-sensing pathways are also associated with human life span and aging. Compelling evidence indicates that the function of the components of the IIS pathway (GH, IGF-1, and insulin) as well as their relevant downstream intracellular effectors (FOXO, AKT, and mTOR) play crucial roles in regulating aging and longevity in both animal models and humans (94). Moreover, premature aging in humans has been reported to be directly linked to metabolic defects (95).

Research has linked circadian rhythm with metabolic regulation. As evidenced by individuals working in the night or rotating shifts and in rodent models of circadian arrhythmia, impairment of the circadian rhythm is significantly associated with reduced longevity and metabolic imbalance (96). Glucose and fatty acid homeostasis are also known to show circadian variation and surgical destruction of the SCN impairs glucose homeostasis (97).

At the molecular level, BMAL1 is one of the crucial components of the circadian clock that controls the cyclic expression of a wide range of metabolic genes involved in glucose, lipid, and cholesterol metabolism (98, 99). The absence of BMAL1 results in not only impairment of its circadian targets but also reduced expression in a great number of metabolic genes, providing direct evidence for BMAL1 being a novel metabolic regulator that couples circadian rhythms and metabolism (100). It has been observed that BMAL1 knock-out mice show insulin resistance (101), diabetes (102), and impaired lipid homeostasis (22). Moreover, increasing evidence indicates that BMAL1 shows linkage to aging and life span through metabolic regulation. Constitutive knockout of BMAL1 in rodent models results in metabolic disturbances and early death. Astrocyte-specific deletion of BMAL1 leads to altered energy balance, impaired glucose homeostasis, acceleration of aging, and reduced lifespan (103).In this review, we summarize the role of BMAL1 in aging-associated metabolic regulation, concerning glucose, fatty acid and amino acid metabolism.

### Glucose Metabolism

The circadian clock controls glucose metabolism by regulating the activity of hormones, metabolic enzymes, and transport systems. Impairment in glucose metabolism, such as reduced glucose-stimulated insulin secretion, insulin resistance, diminished β-cell proliferation and apoptosis, has been associated with asynchrony or deficiencies in circadian clock genes (104). BMAL1 participates in the regulation of glucose homeostasis. CLOCK : BMAL1 heterodimer controls hepatic gluconeogenesis and pancreatic β-cell insulin secretion by mediating the transcription of coactivators that regulate the circadian synthesis of most of the enzymes and hormones involved in glucose homeostasis (105). In skeletal muscle, muscle-specific loss of BMAL1 results in reduced glucose oxidation, disrupted systemic glucose homeostasis, and a diversion of glycolytic intermediates to alternative metabolic pathways (106, 107). Moreover, BMAL1^−/−^ mice have showed reduced circulating insulin (100), which is associated with low levels of one or more proteins of the glucose-sensing and/or insulin secretion pathways caused by the absence of BMAL1 (108).

In addition to hormone regulation of global glucose homeostasis, BMAL1 participates in concrete glucose metabolism processes as follows.

BMAL1 is involved in a variety of energy metabolism processes, especially glycolysis. Increased glycolysis has been associated with cellular senescence. As cell cultures underwent replicative senescence, metabolic profiling showed a significant shift to a more glycolytic state (109). Loss-of function of BMAL1 leads to the disruption of metabolic gene expression associated with a rise of glycolytics. Genetic disruption of BMAL1 results in glycolytic gene expression and glucose tolerance (106). It has been reported that BMAL1-/- liver exhibits increased anaerobic glycolytic gene expression and lactate production under normoxic conditions (110). Importantly, BMAL1 overexpression restrains glycolysis levels. Elevation of BMAL1 in human astrocytes inhibits aerobic glycolysis and lactic acid release by downregulating the expression of HK1 and LDHA (111). Evidence also suggests that BMAL1 exerts a negative regulatory effect on glycolysis through the interaction of calcium binding protein S100A9 (112).

BMAL1 is also involved in pyruvate metabolism as well as the subsequent tricarboxylic acid (TCA) cycle, oxidative phosphorylation (OXPHOS) and ATP production. Pyruvate is a key metabolite at the crossroads of glycolysis and mitochondrial respiration, which determines whether a cell can produce enough energy through glycolysis upon becoming senescent. As senescent cells shift to a glycolytic phenotype, pyruvate and NADH become available substrates for lactate dehydrogenase, producing NAD+ and lactate (113). Activation of BMAL1 inhibits pyruvate dehydrogenase kinase (PDK), resulting in the disinhibition of the pyruvate dehydrogenase complex (PDC). Then PDC drives the conversion of pyruvate to acetyl-coenzyme A (acetyl-CoA) in mitochondria, thus increasing the TCA cycle, OXPHOS and ATP production (114). BMAL1 dysfunction impairs the TCA cycle, OXPHOS and ATP production. Loss of BMAL1 induces significant decreases in metabolites within the TCA cycle and a reduction in TCA cycle intermediates, such as citrate/isocitrate, has been shown in BMAL1 deficient cells (107). Besides, deletion of BMAL1 leads to defects in uncoupling of OXPHOS and glucose-stimulated ATP production (115).

**Figure 3:**
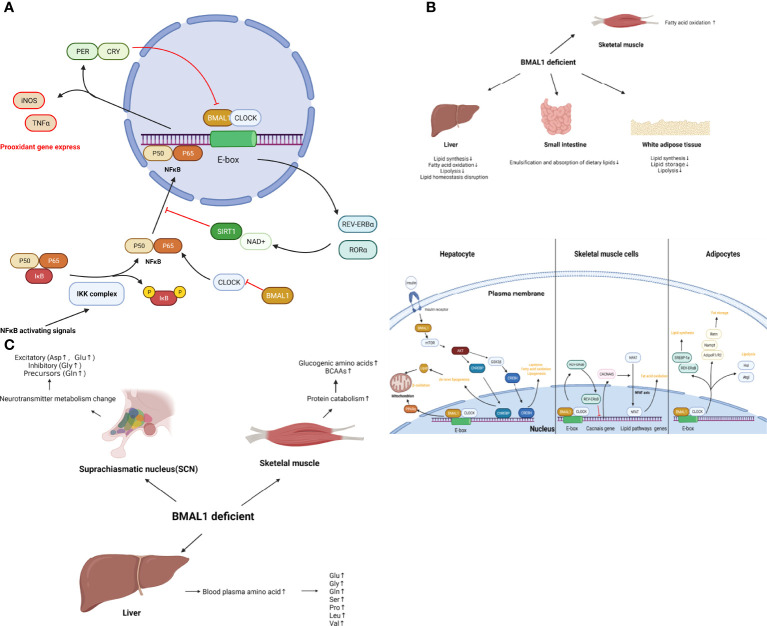
**(A)** The role of BMAL1 in glucose metabolism. BMAL1 controls hepatic glucose metabolism and pancreatic β-cell insulin secretion. In skeletal muscle, muscle-specific loss of BMAL1 results in reduced glucose oxidation. In addition to the hormone regulation of global glucose homeostasis, BMAL1 participates in concrete glucose metabolism processes. BMAL1 restrains glycolysis levels, while activation of BMAL1 inhibits pyruvate dehydrogenase kinase (PDK), resulting in the disinhibition of the pyruvate dehydrogenase complex (PDC). Then PDC drives the conversion of pyruvate to acetyl-coenzyme A (acetyl-CoA) in mitochondria, thus increasing the TCA cycle, OXPHOS and ATP production. Loss-of function of BMAL1 leads to the disruption of the aforementioned process and cellular senescence. **(B)** The role of BMAL1 in lipid metabolism. In the liver, BMAL1 regulates lipid synthesis, fatty acid oxidation, lipolysis and lipid homeostasis. Hepatocyte BMAL1 is required for post-prandial *de novo* lipogenesis through insulin-mTORC2-AKT- ChREBP-lipogenesis. BMAL1 regulates fatty acid oxidation by activating the transcription of the PPARα gene by binding to the E-box within the gene promoter. BMAL1 also regulates homeostasis lipogenesis through CREBH. In white adipose tissue, BMAL1 regulates genes involved in lipogenesis such as SREBP-1a and REV-ERB α, resulting in the promotion of lipid synthesis and accumulation. BMAL1 regulates lipid storage through the expression of Adipoq, Retn, Nampt, AdipoR1 and AdipoR2. BMAL1 regulates lipolysis by directly and rhythmically binding to Eboxes in the promoters of Atgl and Hsl. In skeletal muscle, BMAL1 regulates oxidative capacity and fat oxidation activity through the regulation of Cacnas expression, followed by the NFAT axis. **(C)** The role of BMAL1 in amino acid metabolism. In muscle tissue, loss of function of BMAL1 is associated with protein catabolism. increased glucogenic amino acids (alanine, glutamine, glutamate, glycine, serine, threonine, methionine, proline, and aspartate) as well as BCAAs (leucine, isoleucine, and valine). In the liver, loss of BMAL1 leads to an increase in blood plasma amino acids, such as glutamine, glutamate, glycine, serine, proline, leucine, and valine. In SCN, loss of BMAL1 increases most neurotransmitter amino acid, such as excitatory (Aspartate, Glutamate) or inhibitory (Glycine) and precursors (Glutamine).

Taken together, BMAL1 impairment leads to a glucose metabolism disorder including impaired glucose homeostasis, reduced ATP production, and a shift of metabolic profiling to a more glycolytic state, which is associated with cellular senescence and aging. The role of BMAL1 in glucose metabolism is shown schematically in **Figure 3**.

### Lipid Metabolism

Studies using metabolomics and lipidomics have indicated that hundreds of lipid species are under the regulation of circadian clock in human plasma, including fatty acids, glycerolipids, glycerophospholipids, sphingolipids, sterol lipids and prenol lipids (116). There is emerging evidence that disruption of the circadian clock function results in dysregulation of lipid metabolism, obesity and metabolic diseases (117).

In enterocytes, BMAL1 regulates the emulsification and absorption of dietary lipids. The CLOCK : BMAL1 heterodimer rhythmically activates the small heterodimer partner (Shp), which represses microsomal TAG transfer protein (Mtp), resulting in a diurnal regulation of the lipid transfer and apolipoprotein B-lipoproteins assembly rate (118). Besides, CLOCK : BMAL1 heterodimer regulates the expression of nocturnin (Noc), a crucial metabolic gene for normal lipid absorption and secretion in the small intestine (119).

In liver, BMAL1 regulates lipid synthesis, fatty acid oxidation, lipolysis and lipid homeostasis. BMAL1 is involved in *de novo* lipogenesis. Hepatocyte BMAL1 is required for post-prandial *de novo* lipogenesis through insulin-mTORC2-AKT signaling. By modulating AKT activity in hepatocytes, BMAL1 promotes the efficient conversion of excessive acetyl-CoA into lipids (120). There is also a functional link between BMAL1 and carbohydrate response element binding protein (ChREBP), one of the major lipogenic transcription factors in hepatic lipid biosynthesis. One of the major downstream pathways of BMAL1 in *de novo* lipogenesis may be AKT-ChREBP-lipogenesis (121). BMAL1 deficiency reduces liver AKT activation as well as ChREBP target gene expression which reduces insulin-stimulated *de novo* lipogenesis. Furthermore, BMAL1 regulates fatty acid oxidation. In the liver, all members of the PPAR family, the major regulator of fatty acid oxidation, are diurnally regulated, including PPARα which promotes mitochondrial fatty acid β-oxidation (122). PPARα has been reported to interact with the molecular clock in the liver. The CLOCK : BMAL1 heterodimer activates the transcription of the PPARα gene by binding to the E-box within the gene promoter (123). BMAL1 is crucial for maintaining the PPARα activity as studies have revealed an intimate relationship between the BMAL1-AKT-ChREBP axis-mediated *de novo* lipogenesis and PPARα-induced fatty acid oxidation (121). BMAL1 is also crucial in homeostasis lipid. The core clock oscillator BMAL1 and AKT/glycogen synthase kinase 3β (GSK3β) signaling pathway control cleavage/activation of CAMP-responsive element-binding protein, hepatic-specific (CREBH), a liver-enriched and endoplasmic reticulum (ER)–tethered transcription factor that maintains triglyceride (TG) and fatty acid (FA) homeostasis by regulating expression of the genes involved in lipolysis, fatty acid oxidation, and lipogenesis (124).

In white adipose tissue, BMAL1 participates in the regulation of adipocyte functions and controls lipid metabolism processes such as lipid synthesis, lipid storage and lipolysis. BMAL1 is an important transcription factor in inducing and maintaining specific aspects of the lipid synthesis function, which transcriptionally regulates genes involved in lipogenesis such as SREBP-1a and REV-ERB α (125). In BMAL1 overexpression cells, enhanced expression of SREBP-1a promotes the expression of several factors required for lipogenesis, such as fatty acid synthase (FAS), acetoacetyl-CoA synthetase(AACS), CD36, and HMG-CoA reductase, resulting in the promotion of lipid synthesis and accumulation (125). Enhanced expression of REV-ERBα has been reported to increase the expression of PPARγ target genes, such as aP2 and C/EBPα, thus significantly promoting adipogenesis (126). BMAL1 also regulates lipid storage. There is emerging evidence that BMAL1 deficiency impaired the expression of Adipoq, Retn, Nampt, AdipoR1 and AdipoR2 (127) and reduced the capacity of fat storage in adipose tissue (22), resulting in an increase in the levels of circulating fatty acids and the formation of ectopic fat in the liver and skeletal muscle. Furthermore, BMAL is crucial for lipolysis. Shostak et al. (128) indicated that CLOCK/BMAL1 directly and rhythmically binds to Eboxes in the promoters of Atgl and Hsl, which encode for two lipolysis pacemaker enzymes responsible for over 95% of TG hydrolysis activity (129).

In skeletal muscle, BMAL1 is involved in lipid pathways involved in fatty acid oxidation, lipid synthesis and hydrolysis of TAG (130). Muscle is crucial in lipid metabolism, as it works as a major consumer of lipoprotein-triacylglycerol-derived fatty acid and plasma free fatty acids (FFAs) (131). It has been reported that the level of BMAL1 expression is inversely related to the day-night shift of fat oxidation activity in human skeletal muscles [30]. Wada et al. (132) showed that lack of BMAL1 in murine skeletal muscle displays physiological hallmarks of increased oxidative capacity and fat oxidation activity through regulation of Cacnas expression, followed by activation of calcium—nuclear factor of activated T cells (NFAT) axis, indicating that BMAL1 is a vital regulator of the muscular fatty acid level and involves the control of an oxidative capacity mechanism. Moreover, genome-wide binding results showed that increased expression of major regulatory genes involved in lipid pathways has been found in skeletal muscles of muscle-specific BMAL1 knockout mice, such as mobilizing intracellular lipid stores (Atgl/Pnpla2), channeling fatty acids from lipid droplets to the mitochondria for oxidation (Plin5), fatty acid transport (Fatp-1/Slc27a1), activation of fatty acids to corresponding acyl-CoAs (Acsl1), and finally breakdown (Acadm) and oxidation (Hadha) of fatty acids (133). Therefore, BMAL1 exerts significant tissue-specific differences in regulation of lipid metabolism between muscle and liver.

Collectively, BMAL1 regulates lipid metabolism in different organs, which coordinates the emulsification and absorption of dietary lipids, lipid synthesis, and fatty acid oxidation with daily cycles. Disruption of BMAL1 function leads to dysregulation of lipid metabolism and metabolic disease, indicating the potential role of BMAL1 in accelerated aging. The role of BMAL1 in lipid metabolism is presented schematically in **Figure 3**.

### Amino Acid Metabolism

The impact amino acids have on metabolism and life span has been increasingly investigated, revealing potential targets for aging in humans (134). Some studies have linked BMAL1 with amino acid metabolism.

BMAL1 regulates amino acid metabolism in muscle tissue and muscle tissue plays a highly dynamic role in amino acid metabolism. In the fed state, muscle tissue serves as the main destination for circulating amino acids. In a starvation and insulin deficiency state, muscle tissue serves as the main source of circulating amino acids (135). Loss of function of BMAL1 is associated with protein catabolism. Studies have demonstrated that muscle-specific loss of BMAL1 results in a significant increase of glucogenic amino acids, such as alanine, glutamine, glutamate, glycine, serine, threonine, methionine, proline, and aspartate (133). The BCAAs leucine, isoleucine, and valine, in addition to cysteine, have all been shown to be significantly increased in BMAL1-knockout muscles (133). This study pointed out that muscle-specific loss of BMAL1 can lead to loss of REV-ERBα-dependent repression and persistently increased expression of protein metabolism target genes, which likely accounted for the increased protein turnover (133).

BMAL1 regulates amino acid metabolism in the liver. The liver is the major organ for the catabolism and disposal of amino acids, and postprandial amino acids will mostly be carried to the liver from splanchnic tissues to be catabolized. Therefore, the concentration of amino acids leaving the liver *via* the hepatic vein is determined by liver metabolism. Systemic or liver-specific loss of BMAL1 led to an increase in blood plasma amino acids, such as glutamine, glutamate, glycine, serine, proline, leucine, valine (136).

BMAL1 also has been reported to regulate the amino acid metabolism in the suprachiasmatic nucleus (SCN). Using laser microdissected mouse SCN, significant circadian changes in amino acids were shown, indicating amino acid metabolism is orchestrated by the circadian clock (136). Variations of most amino acid levels in the SCN are independent from circadian metabolism in the liver but are strongly affected by the total loss of BMAL1. Studies have reported that most neurotransmitter amino acids, excitatory (Aspartate, Glutamate) or inhibitory (Glycine) and precursors (Glutamine), increase in the SCN of BMAL1^-/-^ mice (136). These results indicate a link between BMAL1 and neurotransmitter metabolism in the SCN.

Collectively, muscle, liver and SCN show important tissue-specific differences in BAML1-controlled amino acid metabolism. BMAL1 impairment results in protein catabolism in muscle, reduced disposal of amino acids in the liver, and increased blood plasma amino acids, which couples the metabolism disorder with aging. The role of BMAL1 in amino acid metabolism is shown schematically in **Figure 3**.

## Role of BMAL1 in the Genotoxic Stress Response

Genotoxic attacks can originate from extrinsically inflicted radiation damage or chemicals as well as from endogenous sources such as metabolic byproducts or ROS. These factors lead to DNA damage, which can result in either reversible (DNA-damage repair) or irreversible (senescence, transformation, cell death) changes in cell fate (137).

The DNA-damage response (DDR) refers to the mechanisms that detect DNA errors, signal their presence and promote their repair in face of threats posed by DNA damage (138). The DNA repair process involves homologous recombination (HR), nonhomologous end joining (NHEJ), base excision repair (BER), and nucleotide excision repair (NER). DNA double-strand breaks (DSBs) are repaired by HR or NHEJ, whereas single-strand breaks are repaired by BER or NER (139).

The DDR pathway is crucial in DNA repair, which exerts checkpoint functions to block cell cycle progression and prevent the propagation of impaired genetic information to daughter cells. The DDR pathway is mainly activated by the formation of DSBs (5). After DSB formation, the MRE11–RAD50–NBS1 (MRN) complex detects DNA damage sites and recruits ATM and ATR kinases, which phosphorylate the histone variant H2AX. H2AX phosphorylation is required for the assembly of checkpoint proteins and DNA repair factors, such as 53BP1, MDC1/NFBD1, NBS1 and others. γH2AX phosphorylates Chk1 and Chk2 transducer kinases. Activated Chk1 and Chk2 relay the signal to tumour suppressor p53 (140). Activation of p53 leads to transcriptional activation of a wide array of target genes such as the cyclin-dependent kinase inhibitor p21, which in turn results in cell cycle arrest or apoptosis. If repair is achievable, DDR foci disappear, usually within 24 hours, and the cell can resume proliferation (141). However, if DNA damage persists, it causes prolonged DDR signaling and protracted proliferative arrest, which are characteristic of the senescent phenotype and can be a cause of senescence themselves (142).

A multitude of publications link aging with accumulated DNA damage and an impaired DDR pathway. DDR signaling has been considered as a nuclear process as well as extranuclear processes that mediates DNA repair and cell cycle arrest, determines cell fate decisions, reinstates cellular function, counteracts the detrimental consequences of DNA damage accumulation, and extends health span (143). However, persistent DNA damage with DDR defects blocks transcription and replication, which leads to impaired cellular functionality and cellular senescence and apoptosis. As a result, stem cell compartments exhaust, tissues degenerate, and homeostasis declines. In the end, persistent DNA damage contributes to aging phenotypes and to the onset of age-associated diseases (144). For example, DDR defects have been found to display features of premature aging in DDR-defective mice and inherited-DDR-defect patients (138).

**Figure 4:**
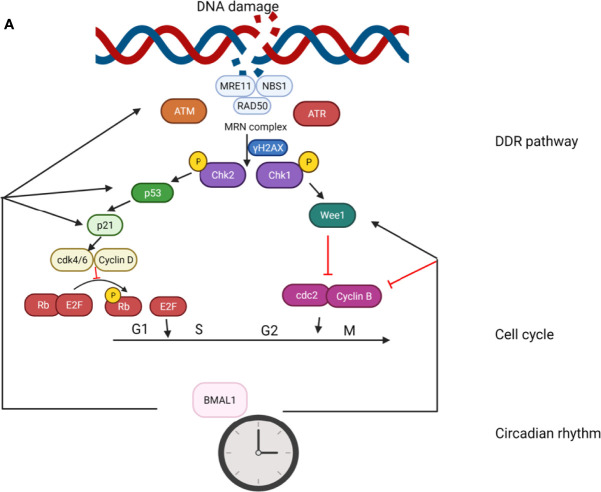
The role of BMAL1 in the genotoxic stress response. The effect of BMAL1 in the genotoxic stress response involves regulation of the DDR pathway and cell cycle (G1/S transition and G2/M transition). BMAL1 regulates the key DSB detection and signaling gene ATM by directly binding to the ATM promoter region. At the bottom of the DDR cascade, BMAL1 regulates p53 by binding to the p53 gene promoter region and transcriptionally activating p53 phosphorylation. BMAL1 regulates some key molecules in the G1/S transition such as p21, Cdk4/6, and Cyclin D. BMAL1 regulates some key molecules in the G2/M transition such as Wee1, cdc2 and Cylin B.

There is emerging evidence that BMAL1 plays crucial roles in the control of response to genotoxic stress, both at the cellular and organismal levels. It has been reported that the expression levels of ATM, phosphorylated Chk2, and p53 are significantly upregulated in BMAL1-overexpressing cells (145). Moreover, depletion of BMAL1 has been noted in cardiomyocytes sensitized to DNA damage and apoptosis in the presence of IR-induced conditions (146). Likewise, a study showed increased sensitivity of BMAL1^–/–^ mice to ultraviolet radiation (UV)-induced DNA damage in the epidermis (147). These studies demonstrate the significance of BMAL1 in the regulation of DDR. In this review, we summarize the effect of BMAL1 in the genotoxic stress response involving the regulation of DDR pathway and cell cycle (G1/S transition and G2/M transition; **Figure 4**.).

### Ataxia-Telangiectasia Mutated Kinase (ATM)

ATM was found as the product of a gene that is lost in Ataxia-Telangiectasia, a rare genetic disease characterized by defects in the immune response, higher incidence of lymphoma development, ataxia and cerebellar neurodegeneration, and premature aging (148). ATM is generally considered a chief mobilizer of the cellular response to DNA damage, which relays a strong, wide-spread signal to numerous downstream effectors in the DDR process (149). ATM is largely linked to senescence. Activated ATM can finely promote senescence and inhibit apoptosis through promoting autophagy and in particular sustains the lysosomal-mitochondrial axis (148).

Compelling evidence points towards regulation between BMAL1 and ATM. At the cellular level, a ChIP assay showed that the key DSB detection and signaling gene, ATM, was bound by BMAL1, indicating that the BMAL1 protein directly binds to the ATM promoter region and its expression level is inversely correlated with the DNA damage levels based on the state of BMAL1 expression (146). Furthermore, BMAL1 exerts its indirect effect on ATM through the repression of its downstream PER1/PER2, which have been reported to interact with ATM in coimmunoprecipitation experiments (150).

### P53

At the end of the DDR cascade, activation of the tumour suppressor p53 plays a key mediator of cellular response to genotoxic stress. Activation of p53 is achieved by increased stability and post-translational modifications of the p53 protein such as phosphorylation, methylation, ubiquitination, and acetylation, which lead to enhanced transcriptional activity of p53 (151). Activation of p53 results in transcriptional activation of a large set of p53 target genes, which in turn mediate cell cycle arrest through the transcriptional induction of cell cycle checkpoints and promote the repair of damaged DNA and subsequent resumption of cell proliferation (152).

As a DDR centerpiece, p53 shows strong interactions with DDR pathway and displays crucial effects on aging and age-associated diseases (153, 154). The p53/p21 signaling pathway is known to be involved in the regulation of cellular senescence, while cellular senescence can also induce p53/p21 activation (155). It has been reported that mice heterozygous for constitutively active p53 display an enhanced aging phenotype (156). Moreover, p53 is also under the regulation of circadian clock (12).

There is emerging evidence that BMAL1 affects the ability of p53 to induce a cell cycle arrest in the genotoxic stress response. Firstly, BMAL1 is considered to be a regulator of the p53 pathway through its control of the transcriptional activity of p53. Mullenders et al. (151) found that BMAL1 is a potential regulator of p53 transcription activity through large‐scale shRNA barcode screening. A later study proved that BMAL1 can directly bind to the p53 gene promoter region, thereby transcriptionally activating p53 phosphorylation to induce apoptosis and cell cycle arrest (157). Secondly, BMAL1 regulates p53 indirectly through the HSR pathway. The Circadian–heat-shock response (HSR) crosstalk commonly occurs in response to genotoxic stress (158). BMAL1 has been reported to be involved in the HSR pathway through BMAL1–HSF1 interaction. Then, the HSR pathway upregulates the p53 pathway HSF1–p53 interaction and subsequent nuclear entry of p53 under genotoxic stress conditions (159). In addition, p53 shows retrograde regulation effects on BAML1. Under stress conditions, stabilized p53 represses the expression of Per2 through the p53-mediated blocking of BMAL1–CLOCK binding to the Per2 promoter, which in turn modulates the expression of BMAL1 and other clock genes (160).

### G1/S Transition

p53 regulates down-stream molecules to maintain the G1/S arrest. This prevents cells from entering the S phase in the presence of DNA damage through inhibition of the initiation of replication. Key factors, such as p21, Cdk2/4/6, Cyclin D/E, and Rb, are involved in the G1/S transition. Phosphorylated p53 induces accumulation of p21, which binds to the Cdk4/Cyclin D complex and prevents it from phosphorylating Rb (161), the key molecule of the G1/S transition, by releasing the E2F transcription factor and promoting transcription of the S-phase genes in its phosphorylated state (162). p21 also binds to, and inactivates, the Cdk2/Cyclin E complex, resulting in the maintenance of the G1/S checkpoint.

The cell cycle inhibitor p21 is considered to be a crucial link between the molecular clock and the cell cycle, has been proven to be under the regulation of BMAL1. p21 mRNA shows a circadian expression in mouse livers and is disrupted in the livers of BMAL1 knockout mice (163), indicating the linkage between BMAL1 and p21. Further studies have proven that loss of BMAL1 can result in a significant decreased expression of p21 in different cells and animal models (164–166).

Studies have indicated that BMAL1 could modulate the p21 expression *via* p53‐dependent or p53‐independent mechanisms. On the one hand, BMAL1 modulates the transcriptional activity of p53 toward its target p21 (151, 157). On the other hand, a multitude of studies have confirmed the fact that BMAL1 can regulate the p21 expression in a p53‐independent manner in different cells (151, 163, 164). Aline Gréchez-Cassiau et al. (163) attributed this manner to regulation of the expression of RORs and REV‐ERBs by BMAL1, which in turn controls the transcription of p21 gene by binding to the ROR response element within its gene promoter.

Studies have also linked the regulation of BMAL1 with other key molecules including Cyclin D (163, 167), Cyclin E (165, 168), and Rb (169). All these studies indicate that BMAL1 plays a crucial role in the G1/S transition.

### G2/M Transition

The G2/M checkpoint of the cell cycle is under tight cellular regulation, which prevents cells from undergoing mitosis in the presence of DNA damage (170). Key factors, such as kinase Wee1, Cdc2, and Cyclin B, are involved in the G2/M transition. In DNA damage, checkpoint kinase Chk1 upregulates Wee1, resulting in the repression of Cdc2/Cyclin B activity and consequent inhibition of the entry into mitosis (171).

One mode of coupling between the cell cycle and BMAL1 is through the kinase Wee1 that inhibits the G2/M transition. CLOCK : BMAL1 heterodimers can induce the transcription of Wee1, which inhibits the kinases Cdc2 through phosphorylation (172). The proliferation rate in hepatocytes of adult BMAL11^-/-^ mice is decreased as a consequence of down-regulation of Wee1 (172), which also highlights the role of BMAL1 in the G2/M transition as well as in accelerated senescence. Likewise, BMAL1 deficiency increased the protein levels of cdc2, Cyclin B1 (173), the critical cellular gatekeepers of the G2/M phase.

Cyclin B1, the critical cellular gatekeeper that regulates cell cycle arrest at the G2/M phase, is also under the regulation of BMAL1. A marked G2/M phase arrest has been noted in BMAL1-overexpressed pancreatic cancer cells, which was linked to a decreased Cyclin B1 expression after Bmal1 overexpression (157). Inactivation of BMAL1 has been shown to lead to delayed G2/M transition in mouse fibroblasts, which was also considered a result of reduced levels of Cyclin B1 (174).

Taken together, these studies demonstrate that BMAL1 is involved in the control of the G2/M transition through regulation of key molecules such as Wee1 and Cyclin B1.

## Conclusion and Prospectives

The circadian rhythm regulates many behavioral, metabolic, and physiological processes, and a multitude of studies has linked the disruption of the circadian rhythm with aging and cellular senescence. BMAL1, the core component of the circadian rhythm, plays a significant role in aging and cellular senescence. In this review, we summarized how BMAL1 exerts its effects on aging through regulation of ROS homeostasis, metabolism, and the genotoxic stress response. In terms of ROS homeostasis, BMAL1 deficiency causes mitochondrial dysfunction and abnormalities in the oxidative-stress-associated pathways (Nrf2 pathway and NFκB pathway), resulting in accumulation of ROS. In terms of metabolic regulation, BMAL1 is involved in glucose, lipid and amino acid metabolism. Disruption of BMAL1 results in insulin resistance, diabetes, hyperlipidemia, increased body fat content and dysregulation of amino acid metabolism. In terms of the genotoxic stress response, BMAL1 participates in the regulation of the DDR pathway and the cell cycle transition. Disruption of BMAL1 causes failure in DNA damage repair and cell cycle arrest. As damaging factors accumulate in BMAL1-deficient organisms, cellular senescence and premature aging eventually arise.

A large body of evidence supports that BMAL1 deficiency impairs physiological processes and causes cellular senescence and premature aging (15). On the other hand, the fact that expression of BMAL1 itself declines in aging indicates that BMAL1 may serve as a potential therapeutic target (16). Therefore, understanding the relationship between BMAL1 and aging will provide a novel therapeutic platform for the study of age-related diseases. Further research is necessary on the genomic and molecular level to establish the role of BMAL1 in aging and cellular senescence. Furthermore, the functions of BMAL1 in cellular senescence are still at a relatively preliminary stage, which calls for more of the translatability to humans of the conclusions on the function of BMAL1 from cell and animal-based experiments.

## Author Contributions

WZ, RT, and YX designed the study and wrote the manuscript. AP reviewed and edited the manuscript. All authors contributed to the article and approved the submitted version.

## Funding

This work was supported by the National Science Foundation of China (No. 82072444, No. 82002313), Department of Science and Technology of Hubei Province (No. 2020BCB004), the National Key Research & Development Program of China (No.2018YFC2001502, 2018YFB1105705), Hubei Province Key Laboratory of Oral and Maxillofacial Development and Regeneration (No.2020kqhm008), the Health Commission of Hubei Province (No. WJ2019Z009), and the Wuhan Union Hospital “Pharmaceutical Technology nursing” special fund (No. 2019xhyn021).

## Conflict of Interest

The authors declare that the research was conducted in the absence of any commercial or financial relationships that could be construed as a potential conflict of interest.

## Publisher’s Note

All claims expressed in this article are solely those of the authors and do not necessarily represent those of their affiliated organizations, or those of the publisher, the editors and the reviewers. Any product that may be evaluated in this article, or claim that may be made by its manufacturer, is not guaranteed or endorsed by the publisher.
